# Effectiveness of community-based management models in chronic obstructive pulmonary disease: a systematic review and meta-analysis

**DOI:** 10.3389/fmed.2025.1584316

**Published:** 2025-06-09

**Authors:** Miao Zhan, Jing Chen, Hongying Zhang

**Affiliations:** ^1^The Affiliated Rehabilitation Hospital of Chongqing Medical University, Chongqing, China; ^2^Department of Breast and Thyroid Surgery, The Second Affiliated Hospital of Chongqing Medical University, Chongqing, China

**Keywords:** chronic obstructive pulmonary disease, community-based management, community-based integrated management, telemedicine management, meta-analysis

## Abstract

**Background:**

Chronic obstructive pulmonary disease (COPD) is a common fatal disease with high morbidity, disability, and economic burden, and it poses a major challenge to global public health. The limitations of the traditional hospital-based management models and the lack of continuous professional guidance and support for people with COPD after discharge have led to repeated acute exacerbations of the disease and high rates of rehospitalization. Community-based management models have received attention because of their convenience, affordability, and accessibility; however, their effectiveness has not been comprehensively and systematically evaluated.

**Methods:**

This study was registered in the International Prospective Register of Systematic Reviews (PROSPERO) and comprehensively searched for randomized controlled trials (RCTs) in the China National Knowledge Infrastructure, Wangfang Data, VIP Database, SinoMed, Cochrane Library, PubMed, and Web of Science from the inception to 6 May 2025. A control group received usual care, and an experimental group received community-based management models (community-based integrated management or telemedicine management, respectively) with an intervention period of >6 months. Two researchers independently used the NoteExpress software for literature management, the Cochrane Risk of Bias Assessment Tool for risk of bias assessment of the included studies, and RevMan5.4.1 for the meta-analysis of outcome indicators.

**Results:**

Thirty-three RTCs, encompassing a cohort of 12,288 people with COPD, were included in this study. The community-based management models demonstrated significant improvements in the 6-min walk test (mean difference [MD] = 39.73; 95% confidence interval [CI, 30.15, 49.32]; *p* < 0.00001) and lung function parameters (forced expiratory volume in the first second/forced vital capacity [FEV1/FVC]: MD = 6.17; 95% CI [4.54, 7.79], *p* < 0.00001; FEV1% predicted: MD = 4.91, 95% CI [3.96, 5.85], *p* < 0.00001). Additionally, it was associated with decreased breathing difficulties (MD = −0.72, 95% CI [−1.23, −0.21], *p* = 0.006) and COPD assessment test (CAT) scores (MD = −4.46, 95% CI [−5.67, −3 0.25], *p* < 0.00001). Telemedicine management also significantly reduced the frequency of acute exacerbations of COPD (MD = −0 0.56; 95% CI [−0.79, −0.32], *p* < 0.00001). Both management models showed comparable effects on the FEV1/FVC ratio, FEV1% predicted, and CAT scores. However, in terms of alleviating dyspnea symptoms—as measured by the modified Medical Research Council scale—community-based integrated management proved superior to telemedicine management (*p* = 0.007). Notably, neither approach demonstrated a statistically significant advantage in improving quality of life among COPD populations, as assessed by the St. George’s Respiratory Questionnaire’s total score (MD = −1.98, 95% CI [−5.02, 1.07], *p* = 0.2).

**Conclusion:**

Community-based management models were significantly better than the usual care in improving exercise tolerance, reducing symptoms, such as dyspnea, and improving lung function in people with COPD. Telemedicine management further reduced the number of acute exacerbations of COPD but did not improve exercise tolerance, and the effect of community-based integrated management on this indicator needs to be verified. Subgroup analyses revealed a significant difference between community-based integrated management and telemedicine management only in improving dyspnea; community-based integrated management was superior to telemedicine management alone. However, neither of the models showed a significant advantage in improving quality of life, suggesting that more comprehensive and precise intervention strategies should be explored in future studies and practice.

**Systematic review registration:**

https://www.crd.york.ac.uk/PROSPERO/view/CRD420251046698, identifier CRD420251046698.

## 1 Introduction

Chronic obstructive pulmonary disease (COPD) is a common fatal disease that is characterized by progressive onset and persistent inflammation. It has become an important challenge to global public health, owing to its high morbidity, disability, and economic burden (1, 2). According to the World Health Organization, COPD will cause 3.5 million deaths in 2021, accounting for approximately 5% of all global deaths, and the disease has become the third leading cause of death worldwide (3). In the European Union, the total direct cost of COPD is estimated to be approximately 56% (€36.8 billion) of the cost of respiratory diseases (4). This shows that effective prevention and control of COPD is essential for the reduction of the global trend of high morbidity and mortality.

In the existing management models of COPD, although hospital clinicians play a crucial role in disease diagnosis, the management of acute exacerbations, and the development of a long-term treatment plan, there are still many limitations to the traditional hospital-centered management approach. Individuals with COPD often lack continuous and systematic professional guidance and support after discharge, leading to persistent deterioration of lung function and repeated acute exacerbations of the disease, which, in turn, causes high rates of rehospitalization (5, 6). Additionally, people’s knowledge and skills of disease self-management are generally low, and their adherence to medication is poor (7, 8), which not only affects the therapeutic effect but also increases the economic burden of the disease.

In recent years, to overcome the barriers of traditional hospital diagnosis and treatment—and to ensure continuity of care for people with COPD beyond hospital settings—community-based management models received increasing attention from scholars. These models have gradually become a focus of study due to their convenient, economic, and accessible characteristics. They extend services related to disease prevention, treatment, rehabilitation, and education at the community level and include approaches such as community-based integrated management and telemedicine management. In community-based integrated management approach, a multidisciplinary team (MDT), including respiratory physicians, nurse specialists, dietitians, psychologists, and pharmacists, has been established to realize offline comprehensive management through respiratory exercises, nutritional support, psychological guidance, health education, drug treatment, and other multidimensional means (9). The telemedicine management approach enables real-time monitoring, remote guidance, and other online continuity management of people with COPD through the integration of the Internet of Things, mobile communication, artificial intelligence, and other technologies (10). However, a comprehensive and systematic assessment of the effectiveness of community-based management models is still lacking, and there are significant gaps in the evidence regarding their generalizability and effectiveness in implementing the full-cycle management of COPD.

Therefore, this study aimed to provide a high-quality, evidence-based foundation for optimizing community-based management models for COPD by systematically evaluating and meta-analyzing existing related studies, thoroughly exploring the effectiveness of two different community-based management models in COPD management, and comprehensively analyzing the roles of community-based management models in improving disease symptoms, improving quality of life, and reducing the frequency of acute exacerbations among COPD populations. This will promote changes in COPD management from hospital-centered care to community-hospital synergistic management models, ultimately improving the long-term prognosis for individuals with COPD and reducing the disease burden on individuals with COPD, their families, and society.

## 2 Method

### 2.1 Protocol registration

This meta-analysis was registered with the International Prospective Register of Systematic Reviews (PROSPERO) (Registration ID: CRD420251046698).

### 2.2 Inclusion and exclusion criteria

The inclusion criteria were as follows:

(i) Adults aged >18 years of age were diagnosed with COPD, and the diagnostic criteria met those of the Global Initiative for Chronic Obstructive Lung Disease (GOLD); (ii) the study type was a randomized controlled trial (RCT) in both Chinese and English languages; (iii) a control group received usual care, while an experimental group received the community-based management models (community-based integrated management or telemedicine management), and the intervention time was >6 months; and (iv) studies reporting at least one of the following outcomes: (1) pulmonary function test results (forced expiratory volume in the first second predicted value [FEV1%], forced expiratory volume in the first second/forced vital capacity [FEV1/FVC]); (2) exercise endurance (6-min walk test [6MWT]); (3) quality of life score (St George’s Respiratory Questionnaire [SGRQ]‘s total score); (4) Modified Medical Research Council Dyspnea Scale (mMRC); (5) COPD assessment test [CAT] score; and (6) number of acute exacerbations. The exclusion criteria were as follows: (1) repeatedly published literature; (2) study locations not limited to the community; (3) study types such as case reports, reviews, meta-analyses, and conference abstracts; (4) unclear intervention times; and (5) inconsistent outcome measures.

### 2.3 Study selection

All RCTs published from China National Knowledge Infrastructure (CNKI), Wangfang Data, VIP Database (VIP), SinoMed, Cochrane Library, PubMed, and Web of Science were retrieved from inception to 6 May 2025. Appendix 1 presents the Chinese and English search strategies used in this study.

### 2.4 Data extraction

Two researchers (MZ and JC) independently screened all retrieved literature based on strictly defined inclusion and exclusion criteria. After completing their reviews, they cross-checked each other’s selections, and, in case of disagreement, they discussed with a third researcher (HYZ). General data, including the authors, time of publication, country, intervention, sample size of experimental and control groups, duration of intervention, and outcome indicators, were extracted.

### 2.5 Risk of bias assessment

NoteExpress software was used to manage the retrieved literature. Two researchers (MZ and JC) independently evaluated the final included studies using the Cochrane Evaluation Tool. After completing checks, they cross-checked each other’s selection, and, in case of disagreement, discussed with a third researcher (HYZ) to reach a final agreement. The main evaluation entries were randomized control, allocation concealment, triple blinding (blinding of participants, researchers, and outcome assessors), completeness of outcome metrics, selective reporting, and other biases.

### 2.6 Statistical analysis

The data extracted from the study were subjected to a meta-analysis using RevMan 5.4.1, and the test level was set at *α* = 0.05. The magnitude of heterogeneity was analyzed using I^2^, and if I^2^ was ≤50%, a fixed-effects model was used, and if I^2^ was >50%, that is, there was clinical heterogeneity (e.g., time of the intervention and target of the intervention), reasons for the heterogeneity were analyzed, and a random-effects model was used. Subgroup and sensitivity analyses were performed to explore sources of heterogeneity. Mean difference (MD) and 95% confidence intervals (CIs) were used as meta-analysis results if the outcome indicator was a continuous variable, and risk ratio and 95% CIs were used as meta-analysis results for dichotomous variables. Funnel plots were used to detect publication bias if the number of studies included in the outcome indicator was >10. Statistical significance was set at a *p*-value of <0.05.

## 3 Results

### 3.1 Study identification and selection

**Figure 1:**
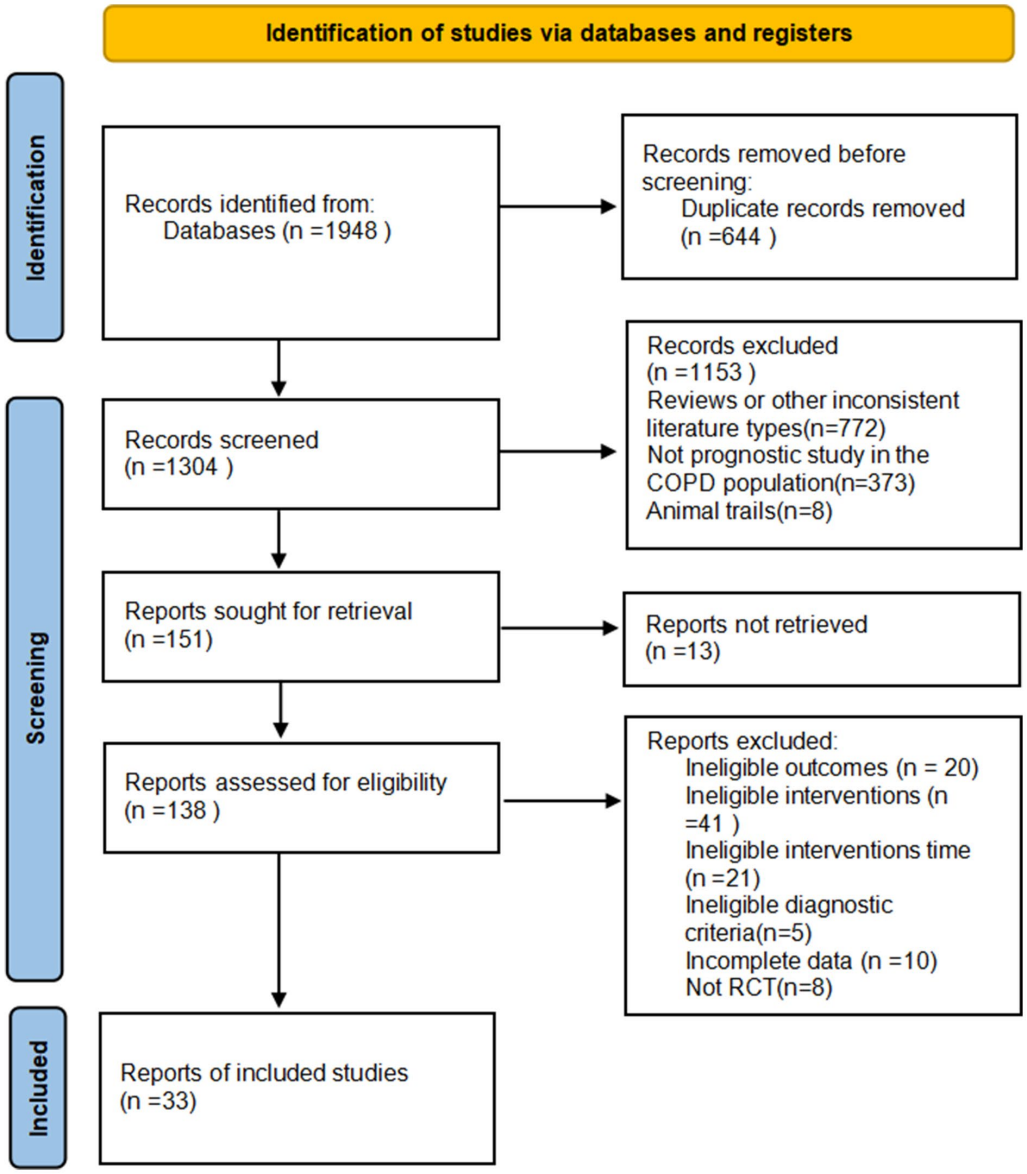
Flow diagram for the selection process of articles.

By searching CNKI (*n* = 242), VIP (*n* = 253), Wanfang database (*n* = 857), PubMed (*n* = 78), Web of Science (*n* = 269), Cochrane Library (*n* = 175), and SinoMed (*n* = 74), a total of 1,948 papers were initially examined and imported into NoteExpress software. After a detailed screening process, 33 RCTs with 12,288 participants who met the inclusion criteria were finally included (Figure 1).

Table 1 presents the basic characteristics of the 33 RCTs (11–43), of which 24 were conducted in China, 1 was a multicenter trial spanning four countries (France, Germany, Italy, and Spain), and the remaining 8 were from Germany, Spain, Australia, Canada, America, and Northern Ireland. Lou had the largest sample size among the included RCTs, with 8,217 participants. A total of 21 studies administered community-based integrated management (including exercise, psychosocial care, nutritional support, and long-term follow-up), while 12 utilized telemedicine management (APP, phone calls, and WeChat).

**Table 1:** Basic characteristics of the included literature (*n* = 33).

Study	Country	Age (mean ± SD)		Sample	Intervention measure		Time	Outcome indicators
		E/C		E/C	E	C		
Longwei Shen 2023 (11)	China	66.84 ± 3.87	66.32 ± 3.46	50/50	Community-based integrated management	Usual care	1 year	②
Na Fan 2020 (12)	China	67.47 ± 7.20	67.39 ± 7.15	49/49	Community-based integrated management	Usual care	6 months	①③
Yan Hong Geng 2022 (13)	China	63.42 ± 5.74	63.52 ± 5.24	34/34	Community-based integrated management	Usual care	1 year	②
Xinghua Guo 2020 (14)	China	64.13 ± 7.86	63.45 ± 6.81	42/42	Community-based integrated management	Usual care	1 year	②
Ling Li 2017 (15)	China	69.3 ± 14.2	70.2 ± 13.9	110/125	Community-based integrated management	Usual care	6 months	②
Ying Gao 2021 (16)	China	67.13 ± 6.87	66.66 ± 9.18	100,100	Community-based integrated management	Usual care	6 months	②③⑥
Qin Wang 2019 (17)	China	68.29 ± 3.27	8.22 ± 3.275	40/40	Community-based integrated management	Usual care	5 months	⑥
Gang Wei 2020 (18)	China	60.2 ± 1.5	60.5 ± 1.6	30/30	Community-based integrated management	Usual care	6 months	②
Nana Liu 2018 (19)	China	64.45 ± 2.21	63.23 ± 2.63	46/45	Community-based integrated management	Usual care	6 months	①③⑤
Kaiming Cui 2020 (20)	China	69.35 ± 8.74	70.12 ± 8.51	70/68	Community-based integrated management	Usual care	1 year	①②③⑥
Weibin Xue 2024 (21)	China	63.25 ± 9.57	62.85 ± 8.97	43/43	Community-based integrated management	Usual care	1 year	①②③⑥
Chao Zhou 2020 (22)	China	69.50 ± 5.72	71.42 ± 6.03	53/53	Community-based integrated management	Usual care	1 year	②③
Caihua Tong 2017 (23)	China	76.4 ± 5.5	77.8 ± 5.7	50/48	Community-based integrated management	Usual care	1 year	②③⑤⑥
Caihua Tong 2016 (24)	China	77 ± 6	76 ± 5	127/125	Community-based integrated management	Usual care	1 year	①②③⑤⑥
Guanghui Pan 2019 (25)	China	79.2 ± 4.3	80.6 ± 3.9	40/40	Community-based integrated management	Usual care	8 months	②③
Behnke 2000 (26)	Germany	64.0 + 1.9	68.0 + 2.2	15/15	Community-based integrated management	Usual care	6 months	①
Ane Arbillaga 2018 (27)	Spain	68 ± 9	69 ± 8	148/132	Community-based integrated management	Usual care	1 year	①⑥
Hermiz 2002 (28)	Australia	67.1	66.7	67/80	Community-based integrated management	Usual care	1 year	④
Kessler 2018 (29)	France, Germany, Italy, and Spain	67.3 ± 8.9	66.6 ± 9.6	157/162	Community-based integrated management	Usual care	1 year	①②③④
Butler 2020 (30)	Canada	68 ± 9	69 ± 9	49/48	Community-based integrated management	Usual care	1 year	①
Lou 2015 (31)	China	61.6 ± 13.5	61.4 ± 13.2	4197/4020	Community-based integrated management	Usual care	4 years	⑥
Robinson 2021 (32)	America	69.2 ± 7.2	70.4 ± 7.3	75/78	Telemedicine management	Usual care	6 months	①④⑤
McDowell 2015 (33)	Northern Ireland	69.8 ± 7.1	70.2 ± 7.4	48/52	Telemedicine management	Usual care	6 months	④⑦
Zanaboni 2023 (34)	America	64.9 ± 7.1	63.5 ± 8.0	40/40	Telemedicine management	Usual care	2 years	①⑤⑥
Jiang 2020 (35)	China	70.92 ± 6.38	71.83 ± 7.60	53/53	Telemedicine management	Usual care	6 months	④⑤⑥
Farmer 2017 (36)	United Kingdom	69.8 ± 9.1	69.8 ± 10.6	110/56	Telemedicine management	Usual care	1 year	④
Lifang Chen 2020 (37)	China	73.6 ± 9.8	72.7 ± 9.1	68/67	Telemedicine management	Usual care	1 year	①③⑥
Quan Yuan 2024 (38)	China	74.6 ± 6.1	75.2 ± 5.9	73/72	Telemedicine management	Usual care	6 months	①②③
Bo Dong 2021 (39)	China	66.9 ± 11.7	67.2 ± 10.8	62/62	Telemedicine management	Usual care	6 months	⑥
Xinying Zhao 2020 (40)	China	67.4 ± 8.1	68.7 ± 5.8	40/37	Telemedicine management	Usual care	1 year	②③⑤⑥⑦
Shaoying Chen 2022 (41)	China	47.83 ± 5.64	46.95 ± 6.07	55/55	Telemedicine management	Usual care	1 year	①②④⑥
Xinhao Cui 2021 (42)	China	72.04 ± 11.13	71.35 ± 10.46	60/60	Telemedicine management	Usual care	6 months	②③⑥
Lei Li 2021 (43)	China	63.57 ± 6.75	62.65 ± 8.55	53/53	Telemedicine management	Usual care	1 year	③⑤⑥⑦

E, experiment group; C, control group; ① 6-min walking test (6MWT); ② forced expiratory volume in the first second/forced vital capacity (FEV1/FVC); ③ forced expiratory volume in 1 s percent predicted (FEV1%predicted); ④ St George’s respiratory questionnaire (SGRQ)’s total score; ⑤ Modified Medical British Research Council (mMRC); ⑥ COPD assessment test (CAT); and ⑦ number of acute exacerbations.

### 3.2 Risk of bias assessment

**Figure 2:**
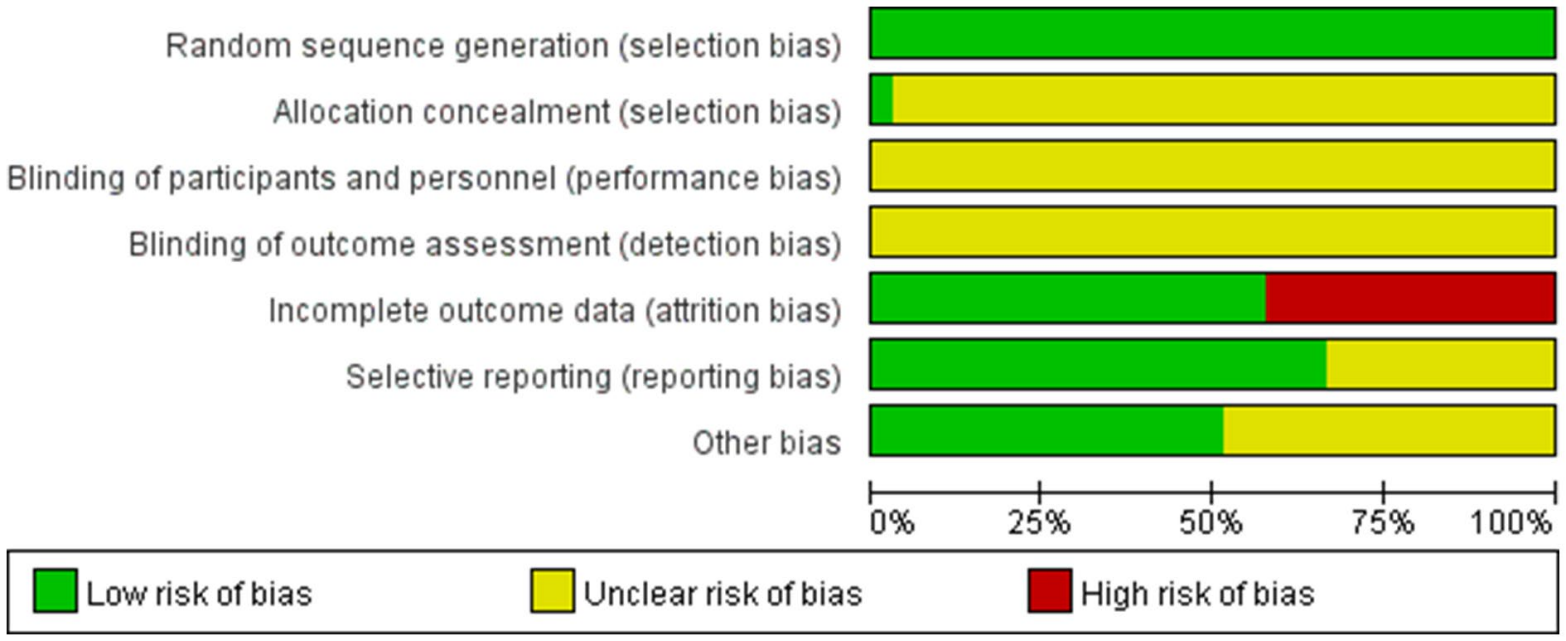
Risk of bias graph in 33 studies.

**Figure 3:**
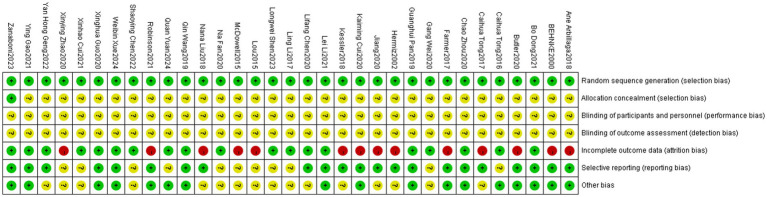
Risk of bias summary assessments for each bias risk item in 33 studies.

Of the 33 included RCTs, only the study by Zanaboni et al. (34) mentioned allocation concealment; however, none of the RCTs mentioned blinding of participants, investigators, or analyzers of results. Fourteen studies (19, 20, 23, 26–33, 35, 36, 40) reported participant dropouts, and the original authors provided explanation for these dropouts, which was primarily due to force majeure related to the natural progression of COPD (e.g., rapid deterioration of the disease requiring treatment termination or death) (Figures 2, 3).

### 3.3 Meta-analysis of outcomes

#### 3.3.1 6MWT

**Figure 4:**
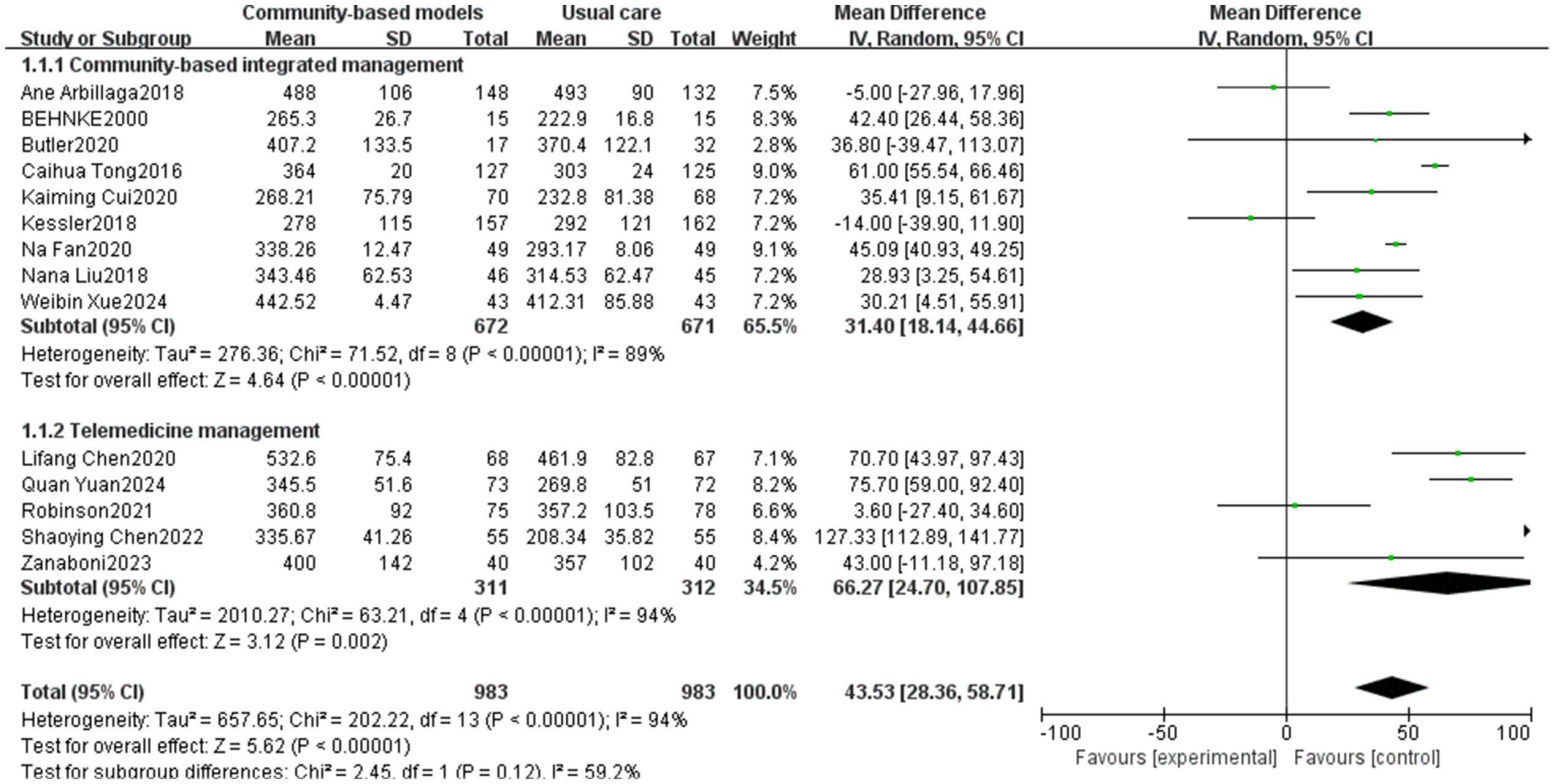
Effect of community-based management models on patients’ 6MWT.

**Figure 5:**
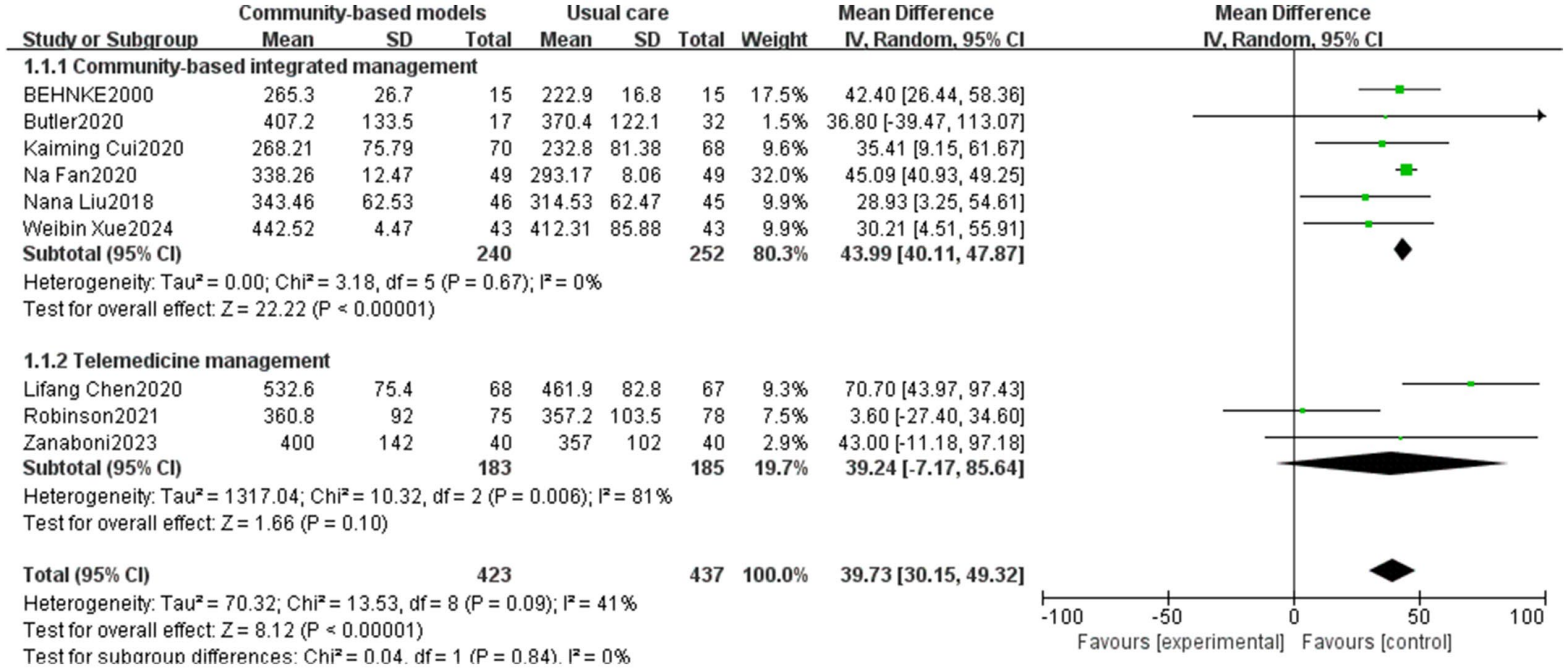
Effect of community-based management models on patients’ 6MWT after sensitivity analysis.

The 6MWT reflects exercise tolerance, cardiorespiratory reserve, disease severity, and prognosis in people with COPD. It is a critical tool for assessing functional status, formulating therapeutic strategies, and monitoring the effects of rehabilitation (44, 45). Fourteen studies (12, 19–21, 24, 26, 27, 29, 30, 32, 34, 37, 38, 41), involving 1,966 people, were included in the analysis (Figure 4). High heterogeneity was observed among the studies (I^2^ = 94%, *p* < 0.00001), and a sensitivity analysis was performed by excluding the studies one by one. After excluding the studies by Ane Arbillaga (27), Caihua Tong (24), Kessler (29), Quan Yuan (38), and Shaoying Chen (41) (Figure 5), the heterogeneity among the remaining nine studies decreased (I^2^ = 41%, p < 0.00001). This reduction could be attributed to the relatively large sample sizes in the studies by Ane Arbillaga, Caihua Tong, and Kessler. Additionally, the studies by Quan Yuan and Shaoying Chen, which focused on telemedicine management, emphasized on respiratory muscle exercise while providing limited psychological guidance and nutritional support, which was the main source of heterogeneity. The results of the meta-analysis, using a random-effects model, demonstrated that the community-based management groups were significantly better than the usual care group in improving the patients’ 6MWT, with a significant difference (MD = 39.73, 95% CI [30.15, 49.32], *p* < 0.00001). However, telemedicine management did not demonstrate a statistically significant effect on improving the 6MWT outcomes (*p* = 0.1). Subgroup analysis revealed that the effects of community-based integrated management and telemedicine management on improving exercise tolerance within COPD populations were essentially the same (*p* = 0.84).

#### 3.3.2 FEV1/FVC

**Figure 6:**
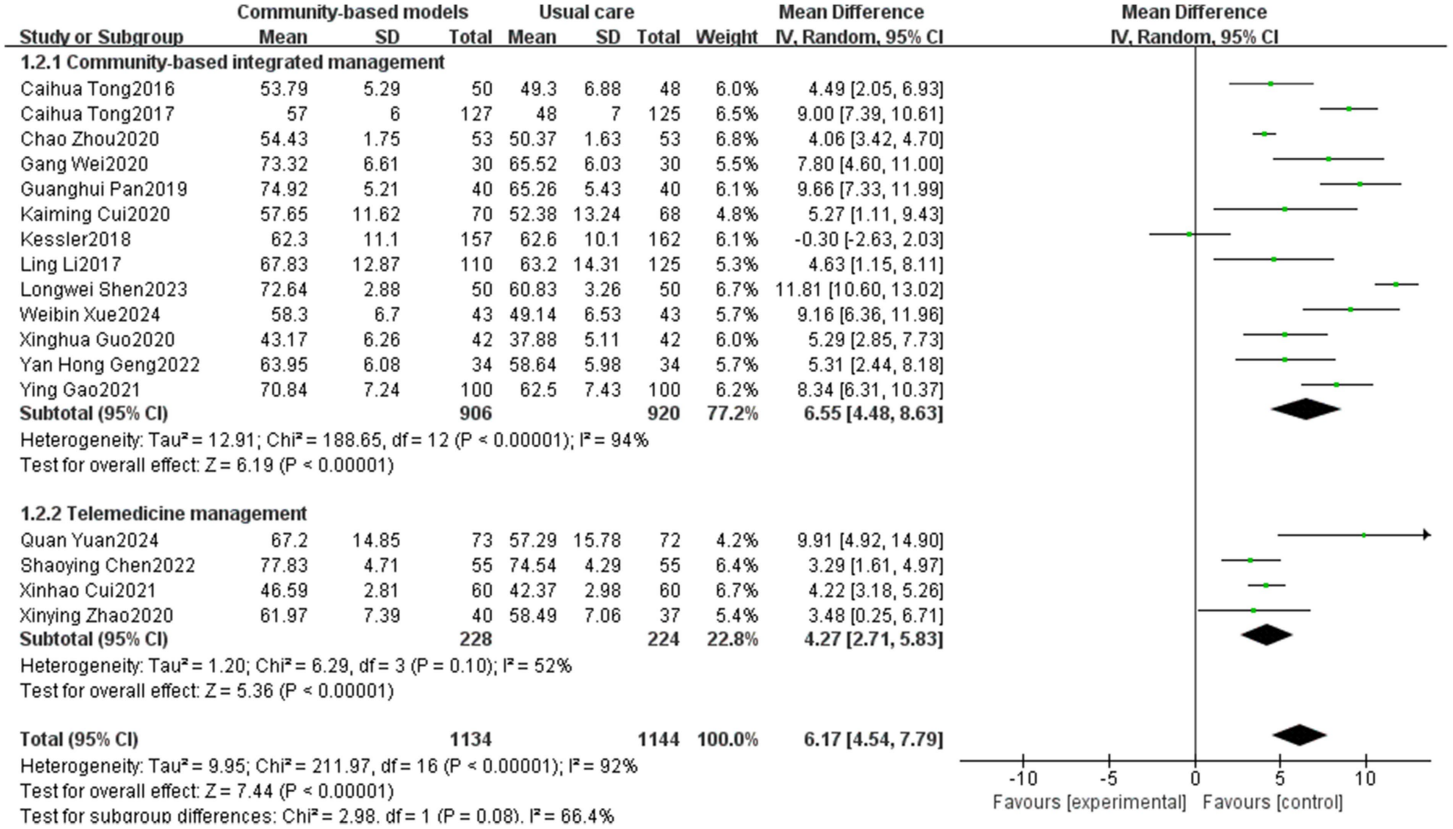
Effect of community-based management models on patients’ FEV1/FVC.

Seventeen studies (11, 13–16, 18, 20–25, 29, 38, 40–42) involving 2,278 participants were included (Figure 6). Owing to the high heterogeneity among the 17 studies (I^2^ = 92%, *p* < 0.00001), the variation may be attributable to the fact that the study by Kessler was a multicenter trial, whereas the others were single-center RCTs. Using a random-effects model, the results showed that FEV1/FVC values were significantly higher in the community-based management groups than those in the usual care group (MD = 6.17, 95% CI [4.54, 7.79], *p* < 0.00001). Subgroup analysis revealed that the community-based integrated management group (MD = 6.55, 95% CI [4.48, 8.63], p < 0.00001) and the telemedicine group (MD = 4.27, 95% CI [2.71, 5.83], *p* < 0.00001) showed significantly greater improvements in FEV1/FVC values than the control group. However, there was no statistically significant difference between the two groups (*p* = 0.08).

#### 3.3.3 FEV1% predicted

**Figure 7:**
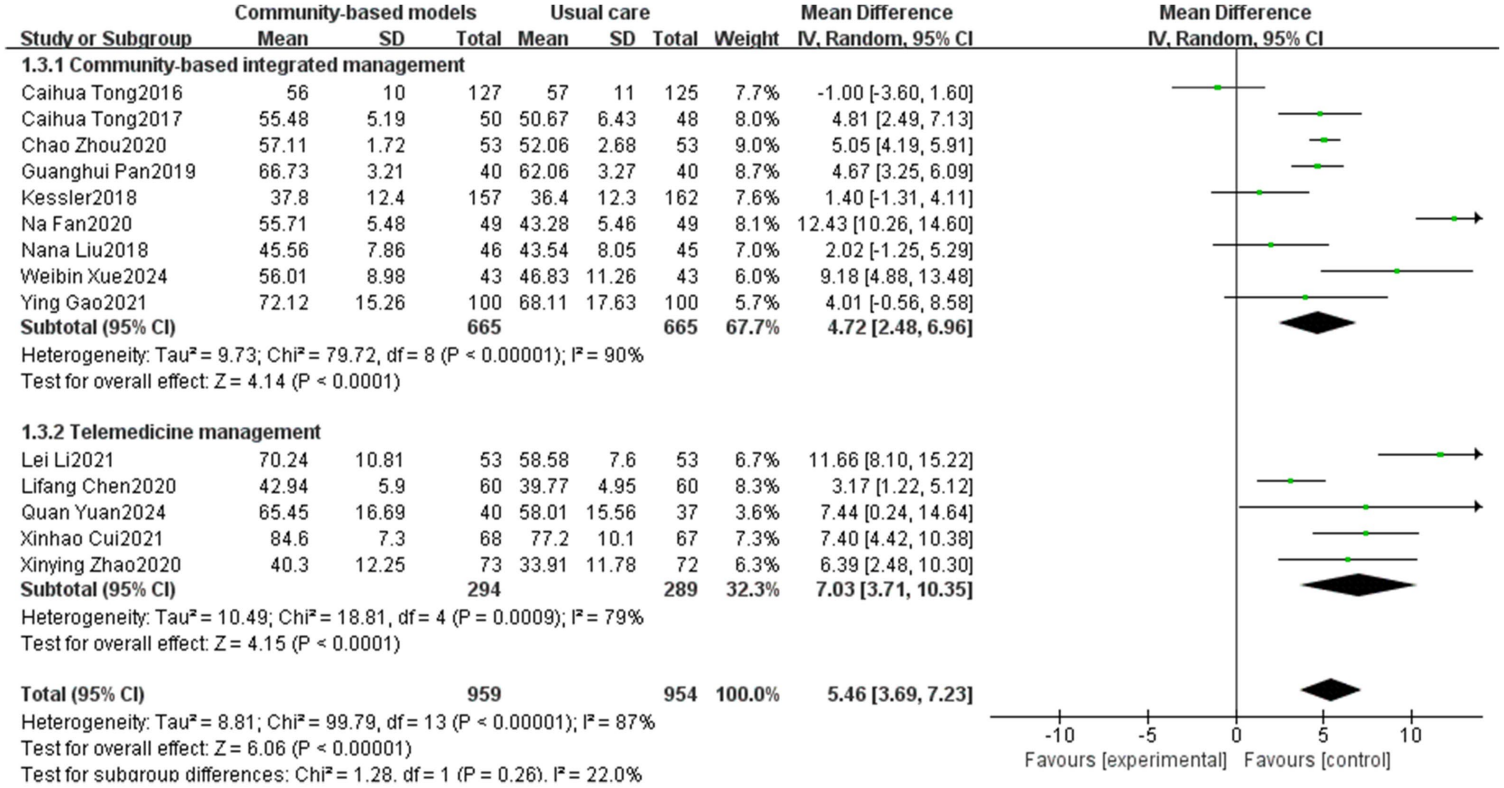
Effect of the community-based management models on FEV1% predicted of patients.

**Figure 8:**
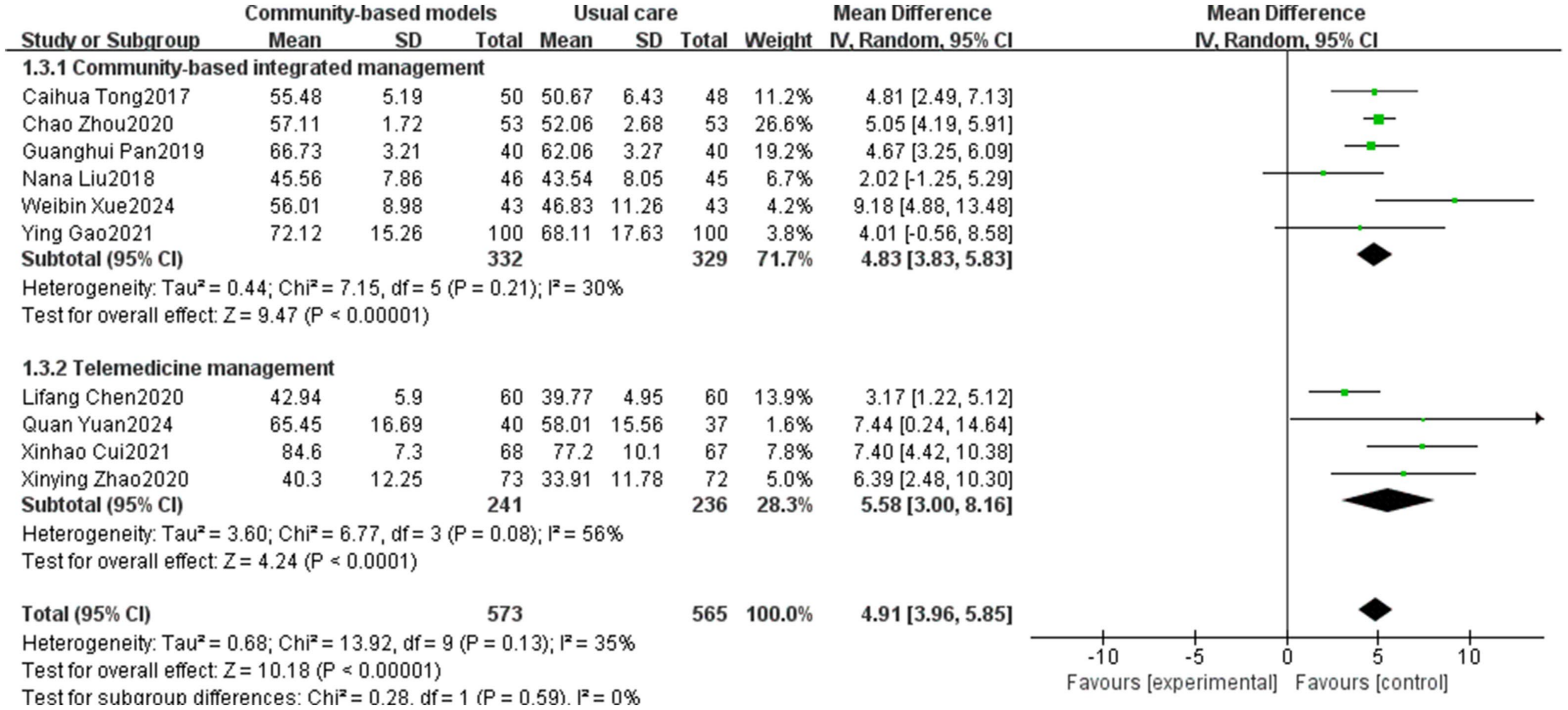
Effect of the community-based management models on FEV1% predicted of patients after sensitivity analysis.

Fourteen studies (12, 16, 19, 21–25, 29, 37, 38, 40, 42, 43), involving 1,913 individuals, were included (Figure 7), with high heterogeneity observed (I^2^ = 87%, *p* < 0.00001). To reduce the heterogeneity of the studies, a sensitivity analysis was conducted to sequentially exclude the studies by Caihua Tong (24), Kessler (29), Na Fan (12), and Lei Li (43). As a result, the heterogeneity among the remaining 10 studies decreased to 35% (Figure 8). The initial high heterogeneity may be due to the relatively large sample sizes in the studies by Caihua Tong and Kessler. Moreover, in the study by Na Fan, restrictions existed in the selection of the study population, for example, the study population should have a COPD disease duration of ≥1 year who were in a clinically stable phase after an acute exacerbation. Additionally, in their study, Lei Li indicated that the study population was required to have access to more than one telemedicine pathway, such as WeChat and the Chronic Lung Disease Manager app, as part of the intervention, which lasted for one year. A meta-analysis using a random-effects model found that community-based management groups were significantly more effective than the usual care group (MD = 4.91, 95% CI [3.96, 5.85], *p* < 0.00001). Subgroup analysis revealed that the effects of community-based integrated management and telemedicine management on improving participant’s FEV1% predicted were essentially the same (*p* = 0.59).

#### 3.3.4 SGRQ’S total score

**Figure 9:**
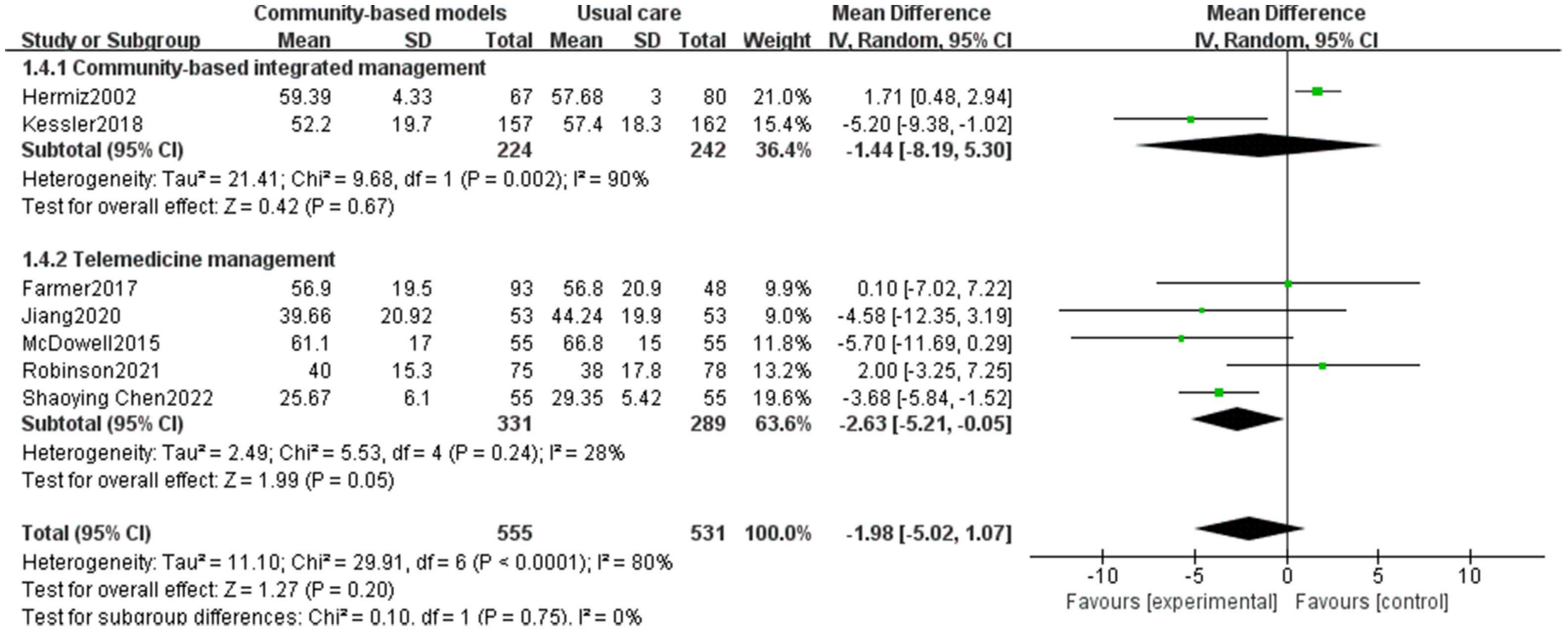
Effect of the community-based management models on patients’ SGRQ’s total score.

The SGRQ’s total score comprehensively reflects the degree of impairment in the quality of life of people with COPD across three dimensions: symptoms, mobility, and psychosocial impact. SGRQ’s total score is an important tool for guiding individualized treatment, assessing the effectiveness of interventions, and predicting prognosis (46). Seven papers (28, 29, 32, 33, 35, 36, 41), including 1,086 people with COPD, were included (Figure 9), with high inter-study heterogeneity (I^2^ = 80%), potentially due to the multicenter nature of Kessler’s study (29), and differences in geographical resources affected the results. The results of the random-effects model showed no statistically significant difference between the community-based management groups and the usual care group in terms of improving the quality of life of people with COPD (MD = −1.98, 95% CI [−5.02, 1.07], *p* = 0.2). Subgroup analysis also revealed no significant difference between the two interventions (*p* = 0.75).

#### 3.3.5 mMRC

**Figure 10:**
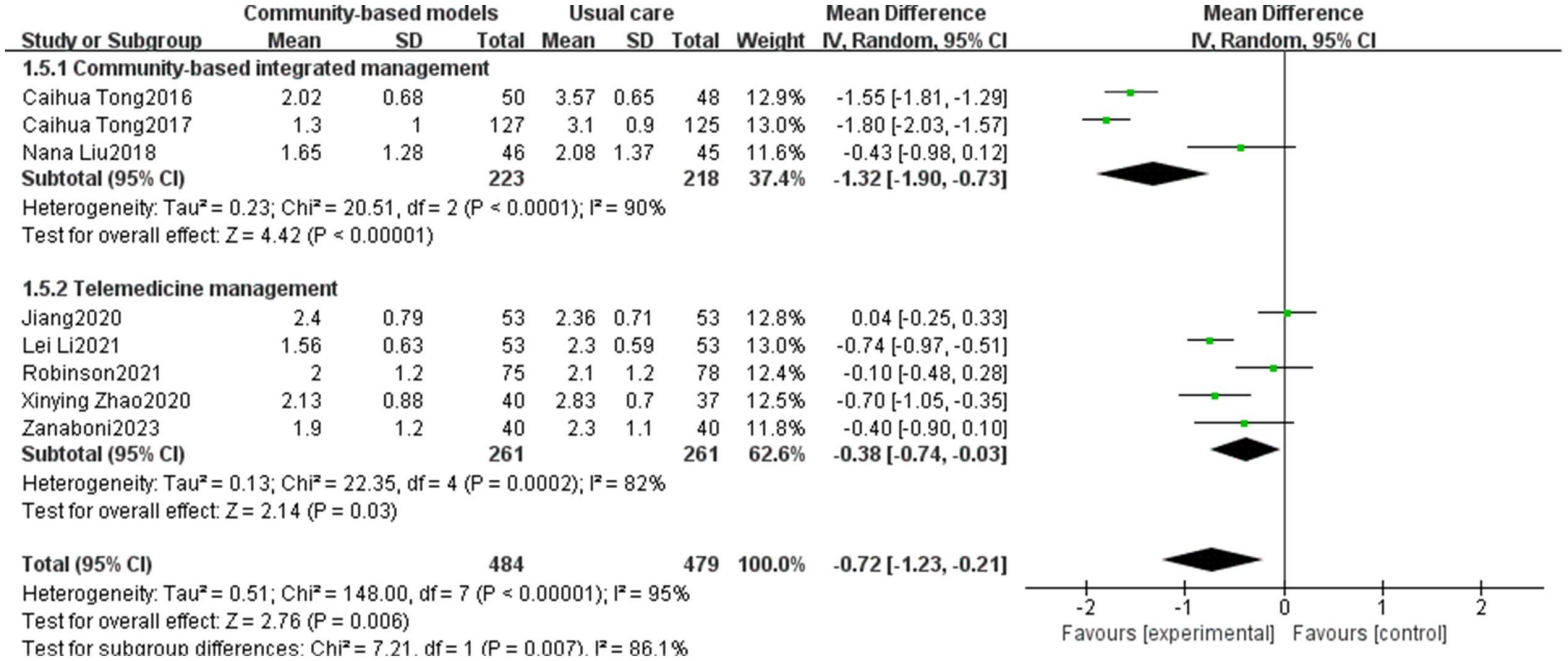
Effect of the community-based management models on the mMRC of patients.

Eight studies (19, 23, 24, 32, 34, 35, 40, 43), involving 963 participants, were included (Figure 10). Inter-study heterogeneity was high (I^2^ = 95%, *p* < 0.00001), and a sensitivity analysis was conducted by excluding studies individually. However, the heterogeneity remained high, which may be attributed to inconsistencies in the intervention time; for example, Caihua Tong’s intervention time was 1 year, whereas Robinson’s intervention time was 6 months. A meta-analysis using the random-effects model revealed that community-based management groups significantly improved dyspnea symptoms among COPD populations (MD = −0.72, 95% CI [−1.23, −0.21], *p* = 0.006). Subgroup analysis exhibited a significant difference between community-based integrated management and telemedicine management in improving dyspnea symptoms, with community-based integrated management being superior to telemedicine management (*p* = 0.007, I^2^ = 86.1%).

#### 3.3.6 CAT scores

**Figure 11:**
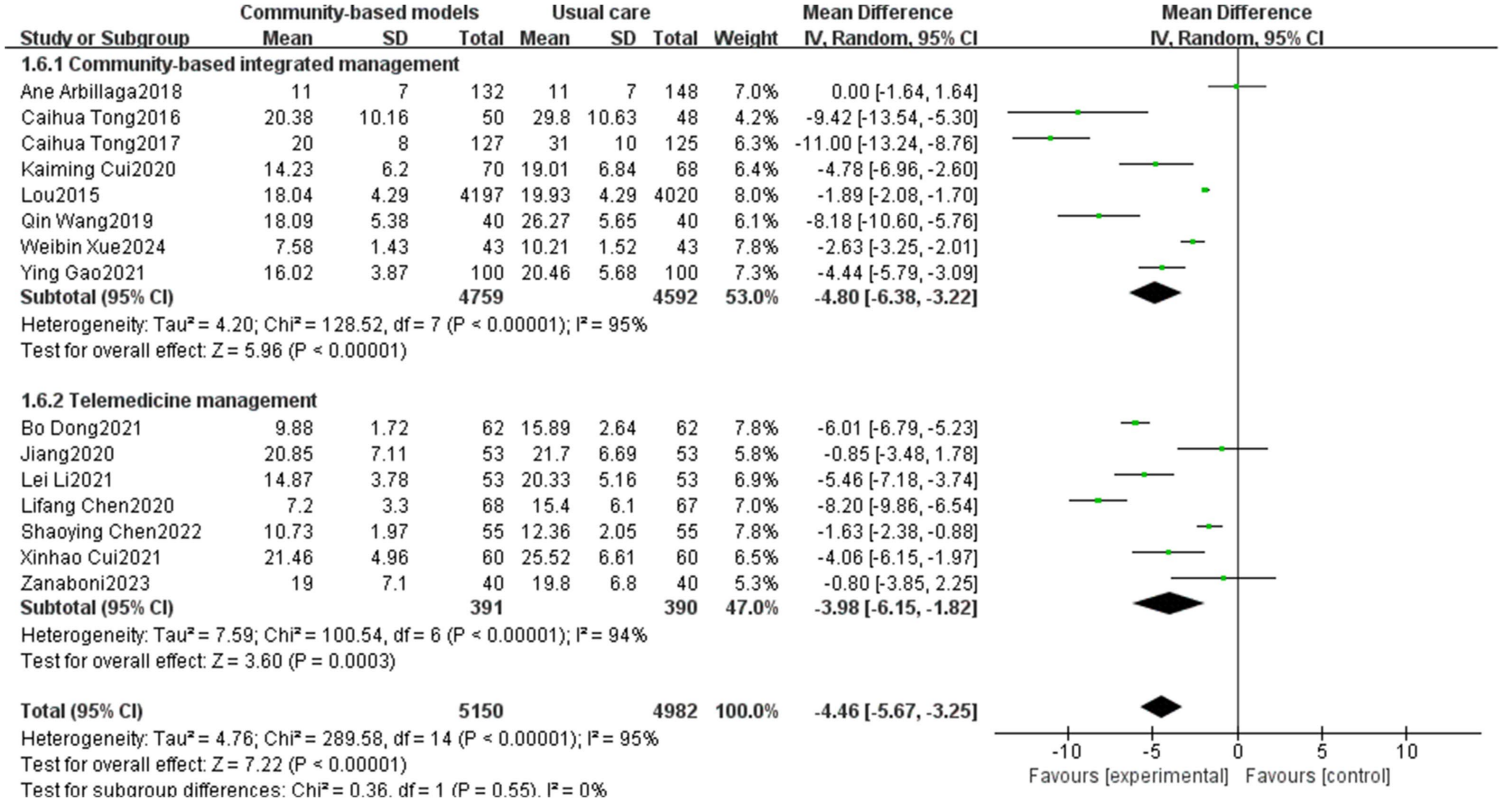
Effect of the community-based management models on the CAT score of patients.

Fifteen papers (16, 17, 20, 21, 23, 24, 27, 31, 34, 35, 37, 39, 41–43), involving a total of 10,132 participants, were included, of which 8 were in the community-based integrated management group and 7 were in the telemedicine group. A high degree of inter-study heterogeneity was observed (I^2^ = 95%, *p* < 0.00001), which may be due to the larger sample size in Lou’s study (4,197/4,020) compared to the other included studies. Sensitivity analysis was performed using the elimination method for individual studies, but heterogeneity remained significantly unchanged, indicating that the results of the studies were more stable. Therefore, a meta-analysis was performed using a random-effects model, and the results revealed that the community-based integrated management group significantly reduced CAT scores in people with COPD (MD = −4.46, 95% CI [−5.67, −3.25], *p* < 0.00001). Subgroup analysis exhibited that the community-based integrated management group performed slightly better than the telemedicine group; however, the difference between subgroups was not statistically significant, indicating no significant difference between the community-based integrated management and telemedicine management in terms of reducing CAT scores (*p* = 0.55, I^2^ = 0%) (Figure 11).

#### 3.3.7 Number of acute exacerbations

**Figure 12:**
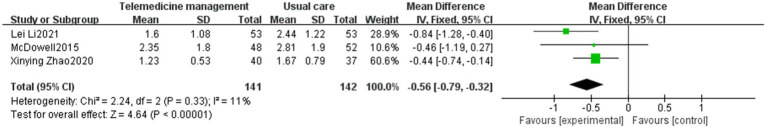
Effect of telemedicine management on the number of acute exacerbations in patients.

Three studies (33, 40, 43), all of which were focused on telemedicine studies involving 283 people, were included, and the results of inter-study heterogeneity analysis were I^2^ = 11% and *p* = 0.33. The number of acute exacerbations of COPD in the telemedicine group was significantly less than that of the usual care group using the fixed-effects model, which was a significant difference (MD = −0.56, 95% CI [−0.79, −0.32], *p* < 0.00001) (Figure 12).

### 3.4 Publication bias

**Figure 13:**
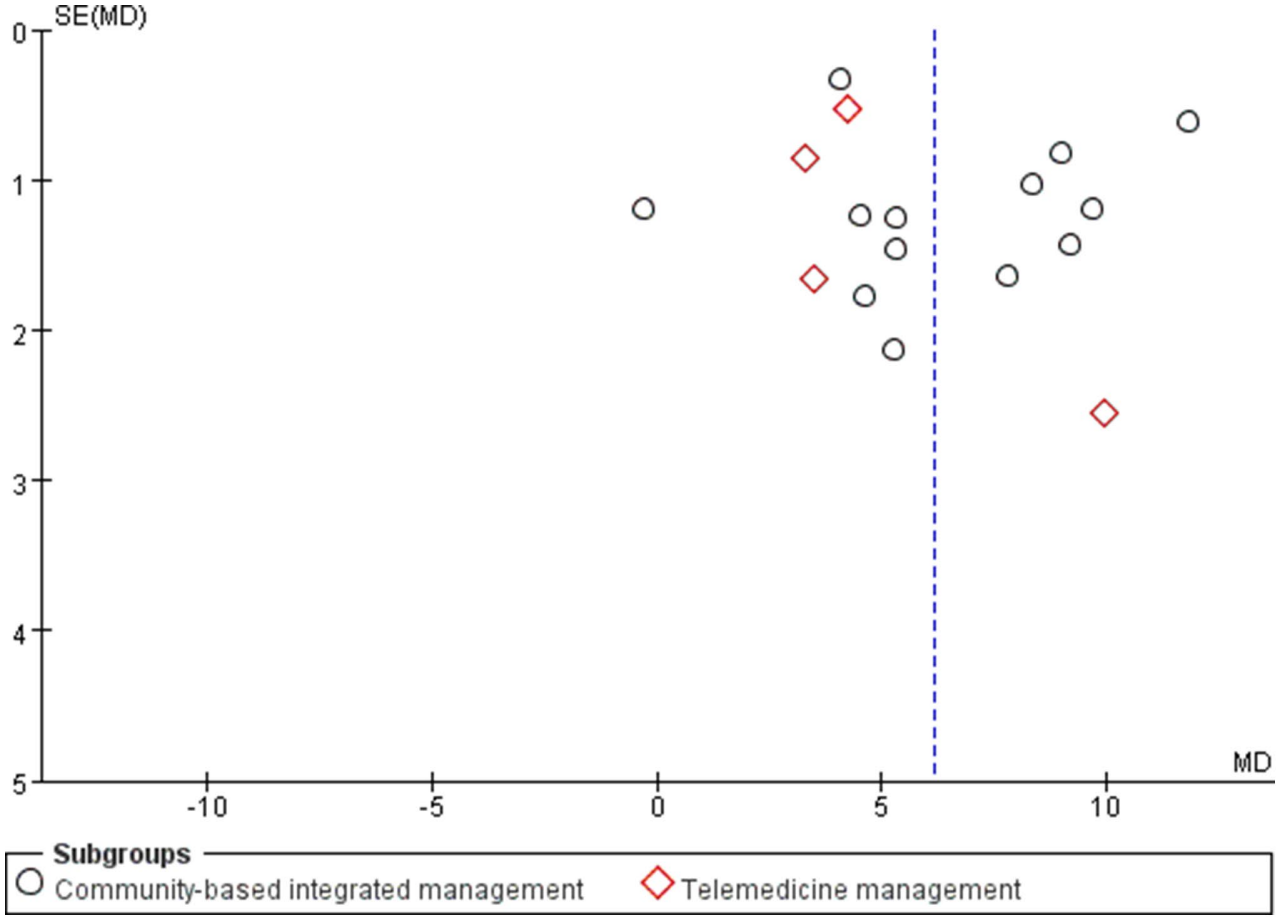
Funnel plot of the community-based management models for patients’ FEV1/FVC.

**Figure 14:**
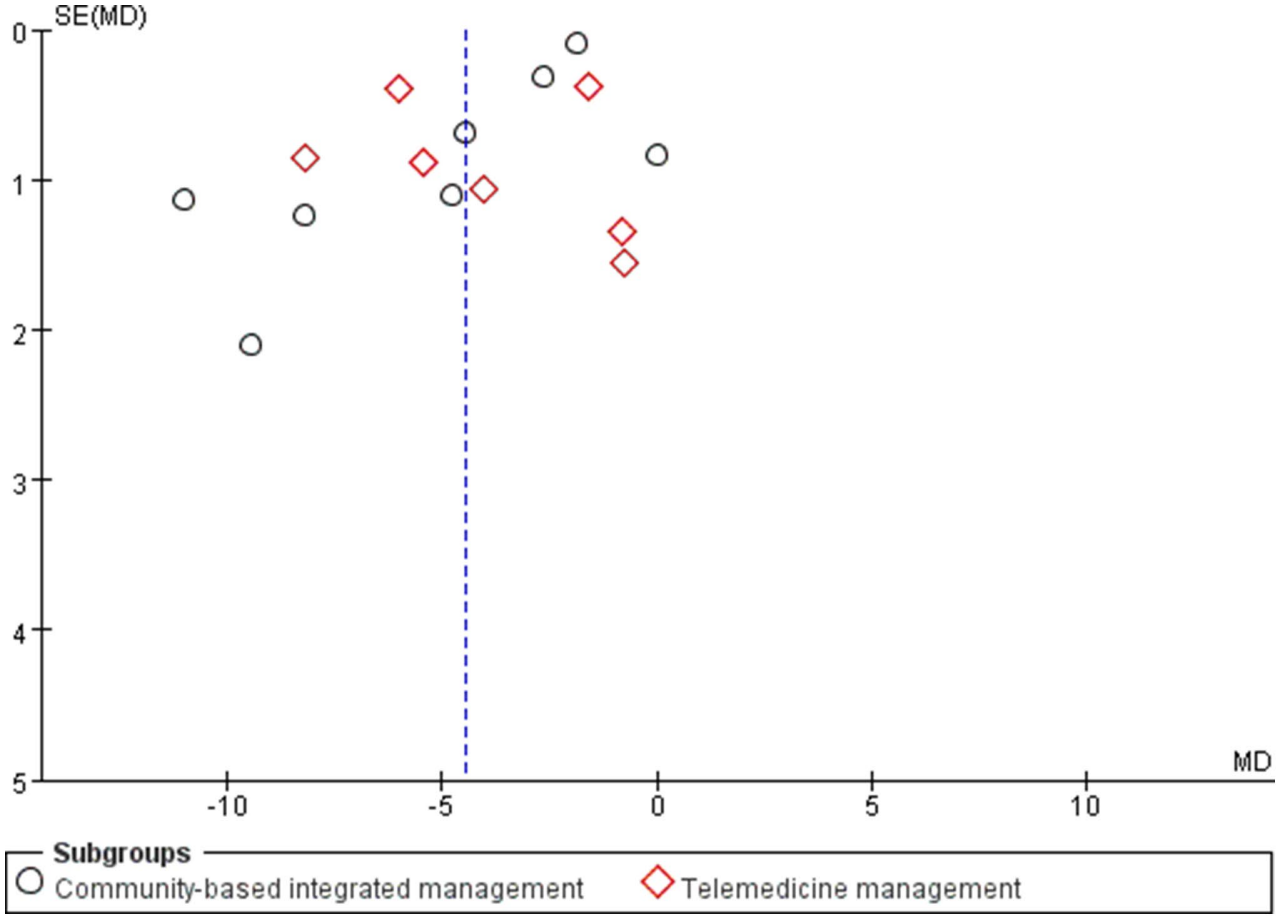
Funnel plot for the CAT score of patients in the community-based management models.

Publication bias was assessed using funnel plots for 17 and 15 studies based on the FEV1/FVC and CAT scores, respectively. In the funnel plot for FEV1/FVC (Figure 13), most of the studies were concentrated at the top; however, the two ends of the funnel plot were asymmetric, indicating the existence of publication or other potential biases. Examples include language bias (only Chinese and English studies were included) and search strategy limitations. For the funnel plot of CAT scores (Figure 14), the results revealed that the funnel plot was asymmetric, which may be attributed to the low methodological quality of the studies. This finding may have led to effect sizes deviating from the true values, with CAT scores in the 15 studies relying on patients’ subjective responses. None of the studies emploted a blinded design, that is, the patients or investigators were aware of the intervention grouping, which may exaggerate or weaken the treatment effect.

## 4 Discussion

### 4.1 Specific analysis of study results

Thirty-three RTCs comprising 12,288 people with COPD were included in this study, and the specific results of the meta-analysis were as follows.

In terms of the 6MWT, this study found that people with COPD in the community-based integrated management group had significantly higher exercise endurance (MD = 43.99, 95% CI [40.11, 47.87], *p* < 0.00001), which could be attributed to the fact that, on the one hand, community-based integrated management integrates multidimensional measures, such as nutritional interventions (e.g., protein supplementation), athletic training, and medication management. In particular, supplementation can increase muscle mass by 1.06%, which directly enhances exercise endurance (47). On the other hand, offline group community exercises can increase social interactions among individuals with COPD, provide additional motivation and support, and help improve the persistence and motivation of pulmonary rehabilitation exercises, further supporting the positive role of community-based integrated management in promoting cardiopulmonary rehabilitation within this population. For FEV1/FVC and FEV1% predicted, which are two important indicators of pulmonary function, the community-based integrated management group demonstrated a significant advantage compared to conventional care (MD = 6.55, 95% CI [4.48, 8.63], *p* < 0.00001; MD = 4.83, 95% CI [3.83, 5.83], *p* < 0.00001; and MD = 4.83, 95% CI [3.83, 5.83], *p* < 0.00001). This is probably due to the early systematic baseline assessment of individuals with COPD (including pulmonary function staging, acute exacerbation risk stratification, comorbidity screening, and health behavior diagnosis) at the community level, which led to the development of an appropriate targeted pulmonary rehabilitation program. Additionally, the advantages of community-based integrated management were validated in terms of alleviating dyspnea symptoms (mMRC classification) and reducing disease burden (the CAT score) (MD = −1.32, 95% CI [−1.9, −0.73], *p* < 0.00001; MD = −4.8, 95% CI [−6.38, −3.22], p < 0.00001).

Telemedicine management was effective in reducing the number of acute exacerbations of COPD (MD = −0.56, 95% CI [−0.79, −0.32]), promoting lung function (FEV1/FVC: MD = 4.27, 95% CI [2.71, 5.83], p < 0.00001, FEV1 predicted: MD = 5.58, 95% CI [3.00, 8.16], *p* < 0.0001), and reducing disease symptoms (mMRC: MD = −0.38, 95% CI [−0.74, −0.03], *p* = 0.03; CAT: MD = −3.98, 95% CI [−6.15, −1.82], *p* = 0.0003), which demonstrated a significant effect; however, in terms of enhancement of exercise endurance (*p* = 0.1), no statistically significant difference was observed. The potential reasons for this lack of difference are as follows: (1) the accuracy of the 6 MWT is dependent on a suitable environment, and remote assessment may result in data bias due to home environment limitations (e.g., space constraints), which affects the interpretation of the results; (2) improvement in exercise tolerance relies on long-term regular exercise training; however, telemedicine may make it difficult for healthcare professionals to effectively monitor participants’ continued participation; and (3) studies have shown that people with COPD are at a higher risk of developing anxiety and depression than the general population (48). Telemedicine may have shortcomings in psychological mediation, such as a lack of effective communication or group support, leading people with COPD to refuse treatment because of emotional barriers. In contrast, community-based integrated management can enhance social support within COPD populations through regular group activities (e.g., group walking and breathing exercises), reduce their sense of social marginalization, and indirectly improve exercise tolerance.

However, this study found that the community-based management models (community-based integrated management and telemedicine management) did not exhibit significant advantages in improving quality of life among COPD participants (SGRQ’s total score). This may be due to the fact that the SGRQ’s total score relies on the subjective assessment of the people with COPD and may not have met the expectations due to differences in the sample population across various studies. In addition, the quality of life of individuals with COPD is related to a variety of complex factors, including psychological status, social support system, and reasonable guidance of medical workers, in addition to the disease itself. It may be difficult to form a one-on-one doctor-patient system to comprehensively solve these difficulties through community-based integrated management or telemedicine interventions, suggesting that more comprehensive and precise intervention strategies should be explored in future studies and clinical practice.

### 4.2 Strengths and limitations of the study

The findings of this meta-analysis are consistent with those of previous systematic evaluations. However, there are also innovative findings. For example, community-based integrated management is consistent with Charlotte’s findings (49) in terms of improving exercise tolerance (6MWT) and dyspnea symptoms (mMRC), but contradicts her findings (49) regarding improving quality of life among COPD populations (SGRQ’s total score). The results of telemedicine were similar to those in the study by Alwashmi (50) in terms of reducing the number of individuals with acute exacerbations. In this study, we conducted a multidimensional expansion based on previous studies on community-based management models. First, we expanded the system of outcome indicators by adding new key physiological and clinical indicators such as FEV1/FVC, FEV1% predicted, and the number of acute exacerbations to systematically verify the validity of the community-based management models. Second, we compared the validity of two community-based management models (community-based integrated management and telemedicine) on different outcome indicators. The results revealed that the two management effects were similar in terms of the 6MWT, lung function (FEV1/FVC and FEV1 predicted), and CAT scores, and it is worth noting that community-based integrated management demonstrated a unique advantage in terms of dyspnea symptom improvement, with a significantly greater effect value for lowering the mMRC classification than that for telemedicine management (MD = −1.32, 95% CI [−1.90, −0.73], *p* < 0.00001), a finding that provides direct evidence for a precise management strategy for dyspnea-dominant people with COPD.

This study has the following limitations:

1. High heterogeneity was observed among studies, towing to several factors: (i) differences in characteristics of the study population, including significant differences in demographic factors of the included studies, such as age, sex, and disease severity, which may affect the generalizability of the intervention effect assessments; (ii) non-uniformity of interventions, with differences in specific intervention content and intervention time of integrated community management (e.g., health education and rehabilitation training) and telemedicine management (e.g., remote platforms and monitoring frequency), limiting comparability of the results; (iii) differences in study design and quality: the included studies contained single- and multi-center trials, with a lack of quality control measures such as experimental participant shedding and blinding application, which increased the heterogeneity of the results; and (iv) although random-effects models or descriptive explanations were used to address high heterogeneity in outcomes (6MWT, FEV1/FVC, FEV1 predicted, SGRQ’s total score, mMRC, and CAT scores), future studies should aim to standardize core parameters of the community-based management models (e.g., the minimum effective period of intervention) to improve the comparability of the findings.
2. Some of the outcomes (SGRQ’s total score) did not reach statistical significance; the difference between community-based integrated management and telemedicine management in improving quality of life and other indicators did not show statistical significance, which may be related to the insufficient sample size and lack of standardization of the intervention protocols.
3. Only articles in Chinese and English were searched, and there may have been language bias. These limitations suggest that the results of this study should be interpreted with caution.

### 4.3 Practical significance of the study and future prospects

This study found that community-based integrated management and telemedicine management have significant clinical value in the management of people with COPD and that the two types of management present complementary advantages. Community-based integrated management significantly improves exercise endurance, lung function, and symptom relief among COPD populations through multidimensional interventions, such as nutrition, exercise, and psychology, implemented through MDT. Offline collective activities in the community can effectively alleviate the psychological burden by strengthening patient–doctor interactions and peer support, which provides a practical basis for primary healthcare institutions to optimize the allocation of resources and perform personalized rehabilitation management. In contrast, telemedicine significantly reduces the risk of acute exacerbation through convenient disease monitoring and symptom management and is particularly suitable for the long-term management of people with COPD in areas with scarce medical resources or limited mobility. Additionally, this study illustrates the strengths of two different management modalities. Community-based integrated management is more suitable for individuals with COPD who need comprehensive physical-psychosocial support, whereas telemedicine has more potential for acute event prevention. This result provides an evidence-based basis for healthcare professionals to choose intervention strategies in the future and helps promote the transition of the COPD management model from the traditional single-care to full cycle and precision.

Although this study demonstrates the core advantages of the two community-based management models, there are still limitations to the improvement of quality of life within COPD populations, suggesting the need for further exploration of multifactorial synergistic intervention pathways. Moreover, studies have shown that community-based management models still have limitations, such as unequal distribution of medical resources, unstandardized follow-up management, and lagging information technology (51, 52). In the future, with the continuous development of the medical-industrial cross-combination field and continuous innovation in healthcare models, it is expected that community-based integrated management and telemedicine management will become further integrated. Through interdisciplinary cooperation, a standardized, normative, and replicable COPD community-based management program will be established, promoting the innovation of “hospital-community-family” chronic disease management models.

## 5 Conclusion

The results of this study showed that (1) the community-based management models were significantly better than usual care in improving exercise tolerance (6MWT), reducing dyspnea and other symptoms (mMRC and CAT), and improving lung function (FEV1/FVC and FEV1% predicted) in people with COPD; (2) telemedicine further reduced the number of acute exacerbations of COPD but did not improve exercise tolerance, and the effect of community-based integrated management on this indicator remains to be validated; (3) subgroup analyses revealed a significant difference between community-based integrated management and telemedicine management only in improving dyspnea (mMRC); community-based integrated management was superior to telemedicine management; and (4) the available evidence fails to support the idea that the community-based management models improve quality of life within COPD populations (SGRQ’s total score).

## Data Availability

The original contributions presented in the study are included in the article/Supplementary material; further inquiries can be directed to the corresponding authors.
