# Supplementary material for: Effectiveness of community-based management models in chronic obstructive pulmonary disease: a systematic review and meta-analysis
Source: Front Med (Lausanne). 2025 Jun 9;12:1584316. doi: 10.3389/fmed.2025.1584316 (PMC12183027; doi:10.3389/fmed.2025.1584316)
Supplement: Supplementary file 1 [file Data_Sheet_1.docx]

| Database | Search strategy |
| --- | --- |
| China National Knowledge Infrastructure (CNKI) | （慢性阻塞性肺疾病 + 慢阻肺 + COPD）*  （综合护理 + 多学科团队 + 协作护理 + 综合干预 + 社区管理 + 远程医疗 + 远程监测 + 远程健康 + 移动健康）*（社区 + 家庭） |
| Wangfang Data | ((主题=(（慢性阻塞性肺疾病 OR 慢阻肺 OR COPD）)) AND 主题=(（综合护理 OR 多学科团队 OR 协作护理 OR 综合干预 OR 社区管理 OR 远程医疗 OR 远程监测 OR 远程健康 OR 移动健康）)) AND 主题=(（社区 OR 家庭）) |
| VIP Database (VIP) | [((((题名或关键词=慢性阻塞性肺疾病 OR 题名或关键词=慢阻肺) OR 题名或关键词=COPD) AND ((((((((题名或关键词=综合护理 OR 题名或关键词=多学科团队) OR 题名或关键词=协作护理) OR 题名或关键词=综合干预) OR 题名或关键词=社区管理) OR 题名或关键词=远程医疗) OR 题名或关键词=远程监测) OR 题名或关键词=远程健康) OR 题名或关键词=移动健康)) AND (题名或关键词=社区 OR 题名或关键词=家庭))](http://pffgx910c42d660f143f8houtuidoutuid4a81.zzzz.cwlib.cqmu.edu.cn/Qikan/search/index?LngMySearHistoryIdGuid=df93ac7f-edb6-4fb6-af19-b5b57751f32c&from=Qikan_Article_History) |
| Sinomed | (("慢性阻塞性肺疾病"[常用字段] OR "Chronic Obstructive Pulmonary Disease"[常用字段] OR "慢阻肺"[常用字段] OR "COAD"[常用字段] OR "COPD"[常用字段] OR "慢性气道阻塞性疾病"[常用字段] OR "慢性阻塞肺疾病"[常用字段] OR "慢性气道阻塞"[常用字段] OR "慢性气流阻塞"[常用字段] OR "肺疾病, 慢性阻塞性"[主题词]) OR ("慢阻肺"[常用字段] OR "慢性阻塞性肺疾病"[常用字段] OR "Chronic Obstructive Pulmonary Disease"[常用字段] OR "COAD"[常用字段] OR "COPD"[常用字段] OR "慢性气道阻塞性疾病"[常用字段] OR "慢性阻塞肺疾病"[常用字段] OR "慢性气道阻塞"[常用字段] OR "慢性气流阻塞"[常用字段] OR "肺疾病, 慢性阻塞性"[主题词]) OR ("COPD"[常用字段] OR "慢性阻塞性肺疾病"[常用字段] OR "Chronic Obstructive Pulmonary Disease"[常用字段] OR "慢阻肺"[常用字段] OR "COAD"[常用字段] OR "慢性气道阻塞性疾病"[常用字段] OR "慢性阻塞肺疾病"[常用字段] OR "慢性气道阻塞"[常用字段] OR "慢性气流阻塞"[常用字段] OR "肺疾病, 慢性阻塞性"[主题词])) AND ("综合护理"[常用字段] OR "多学科团队"[常用字段] OR "协作护理"[常用字段] OR "综合干预"[常用字段] OR "社区管理"[常用字段] OR ("远程医疗"[常用字段] OR "远程医学"[常用字段] OR "Telemedicine"[常用字段] OR "移动医疗"[常用字段] OR "在线医疗"[常用字段] OR "远程医学"[主题词]) OR "远程监测"[常用字段] OR "远程健康"[常用字段] OR "移动健康）"[常用字段]) AND ("（社区"[常用字段] OR "家庭）"[常用字段]) |
| Cochrane Library | (Chronic Obstructive Airway Disease OR Chronic Obstructive Lung Disease OR COAD OR Chronic Obstructive Pulmonary Disease OR COPD OR Chronic Airflow Obstruction) in Title Abstract Keyword AND (Integrated care OR Multidisciplinary team OR Collaborative care OR integrated intervention OR Community-based management OR Telemedicine OR Telemonitoring OR Telehealth OR mHealth) in Title Abstract Keyword AND (Community-based OR home-based) in Title Abstract Keyword - (Word variations have been searched) |
| Pubmed | (((Chronic Obstructive Airway Disease OR Chronic Obstructive Lung Disease OR COAD OR Chronic Obstructive Pulmonary Disease OR COPD OR Chronic Airflow Obstruction)) AND ((Integrated care OR Multidisciplinary team OR Collaborative care OR integrated intervention OR Community-based management OR Telemedicine OR Telemonitoring OR Telehealth OR mHealth))) AND ((Community-based OR home-based)) |
| Web of Science | ((ALL=(Chronic Obstructive Airway Disease OR Chronic Obstructive Lung Disease OR COAD OR Chronic Obstructive Pulmonary Disease OR COPD OR Chronic Airflow Obstruction)) AND ALL=(Integrated care OR Multidisciplinary team OR Collaborative care OR integrated intervention OR Community-based management OR Telemedicine OR Telemonitoring OR Telehealth OR mHealth)) AND ALL=(Community-based OR home-based) |
